# Positive Attributes Buffer the Negative Associations Between Low Intelligence and High Psychopathology With Educational Outcomes

**DOI:** 10.1016/j.jaac.2015.10.013

**Published:** 2016-01

**Authors:** Mauricio Scopel Hoffmann, Ellen Leibenluft, Argyris Stringaris, Paola Paganella Laporte, Pedro Mario Pan, Ary Gadelha, Gisele Gus Manfro, Eurípedes Constantino Miguel, Luis Augusto Rohde, Giovanni Abrahão Salum

**Affiliations:** a Hospital de Clínicas de Porto Alegre, Porto Alegre, Brazil (HCPA) and Universidade Federal do Rio Grande do Sul, Porto Alegre, Brazil (UFRGS); b Section on Bipolar Spectrum Disorders, Intramural Research Program, National Institute of Mental Health and National Institutes of Health, Bethesda, MD; c King’s College London, Institute of Psychiatry, London; d Universidade Federal de São Paulo, São Paulo, Brazil (UNIFESP) and the National Institute of Developmental Psychiatry for Children and Adolescents, São Paulo (INCT-CNPq); e HCPA, UFRGS, and INCT-CNPq; f UNIFESP, INCT-CNPq, and the Institute of Psychiatry, Universidade de São Paulo (USP); g HCPA, UFRGS, INCT-CNPq, and USP; h UFRGS and INCT-CNPq

**Keywords:** noncognitive skills, youth strengths inventory, interaction, school

## Abstract

**Objective:**

This study examines the extent to which children’s positive attributes are distinct from psychopathology. We also investigate whether positive attributes change or “buffer” the impact of low intelligence and high psychopathology on negative educational outcomes.

**Method:**

In a community sample of 2,240 children (6–14 years of age), we investigated associations among positive attributes, psychopathology, intelligence, and negative educational outcomes. Negative educational outcomes were operationalized as learning problems and poor academic performance. We tested the discriminant validity of psychopathology versus positive attributes using confirmatory factor analysis (CFA) and propensity score matching analysis (PSM), and used generalized estimating equations (GEE) models to test main effects and interactions among predictors of educational outcomes.

**Results:**

According to both CFA and PSM, positive attributes and psychiatric symptoms were distinct constructs. Positive attributes were associated with lower levels of negative educational outcomes, independent of intelligence and psychopathology. Positive attributes buffer the negative effects of lower intelligence on learning problems, and higher psychopathology on poor academic performance.

**Conclusion:**

Children’s positive attributes are associated with lower levels of negative school outcomes. Positive attributes act both independently and by modifying the negative effects of low intelligence and high psychiatric symptoms on educational outcomes. Subsequent research should test interventions designed to foster the development of positive attributes in children at high risk for educational problems.

Educational attainment in childhood is a powerful predictor of economic success, health, and well-being later in life.^1^, ^2^, ^3^ Both intelligence^4^ and psychiatric symptoms^5^, ^6^ influence an individual’s performance in educational settings. However, recent econometric studies also highlight the impact of positive attributes—such as being eager to learn, affectionate, and caring—on educational attainment.^7^, ^8^, ^9^, ^10^ Although research has begun to examine the role of positive attributes on determining education outcomes,^11^, ^12^ major questions remain.

First, it is important to determine whether positive attributes are a distinct construct, separable from the absence of psychiatric symptoms.^11^ Economic studies cannot answer this question because they do not include measures of psychopathology. The few available studies in psychiatry^11^, ^12^ support the independent contributions of positive attributes and psychiatric symptoms in predicting the subsequent development of psychiatric illness. However, the distinction between positive attributes and psychiatric symptoms has not been examined psychometrically.

Second, if positive attributes are indeed distinct from the absence of psychiatric symptoms, it is important to investigate interactions between these 2 constructs and intelligence in predicting educational outcomes. Consistent with economic theories of human development, evidence suggests that positive attributes and intelligence may interact in predicting educational outcomes, such as school graduation by age 30 years.^1^, ^13^ However, no studies investigate interactive effects between positive attributes and psychopathology on educational outcomes. Specifically, it is important to ascertain whether positive attributes buffer the negative impact of low intelligence and high psychiatric symptoms on educational outcomes. If positive attributes have such buffering properties, then facilitating their emergence might improve outcomes in children who are at risk for adverse educational outcomes because of psychiatric symptoms or low intelligence.

Here we aim to investigate the following: the discriminant validity of the constructs of positive attributes and psychiatric symptomatology in children; and whether positive attributes are independently associated with educational outcomes and/or whether they buffer associations between low intelligence and negative educational outcomes, and between high psychiatric symptoms and negative educational outcomes. First, we predict that positive attributes are empirically discriminable from psychiatric symptoms. Second, we predict that positive attributes are associated with lower levels of negative educational outcomes independent of intelligence and psychopathology, and through interactions with low intelligence and high levels of psychiatric symptoms that buffer the impact of these 2 variables on negative educational outcomes.

## Method

### Participants

We used data from a large, school-based, community study that obtained psychological, genetic, and neuroimaging data and was designed to investigate typical and atypical trajectories of psychopathology and cognition over development.^14^ The ethics committee of the University of São Paulo approved the study. Written consent was obtained from parents of all research participants, and verbal assent was obtained from the children.

The study included screening and assessment phases. The screening phase of the study included children from 57 public schools in São Paulo and Porto Alegre, Brazil. In Brazil, on specified registration days, at least 1 caregiver is required to register each child for compulsory school attendance. All parents and children who presented at the selected schools were invited to participate. Families were eligible for the study if the children were registered by a biological parent capable of providing consent and information about the child’s behavior, were between 6 and 12 years of age, and remained in the same school during the study period.

We screened 9,937 parents using the Family History Survey (FHS).^15^ From this pool, we recruited 2 subgroups: 1 subgroup randomly selected (n = 958), and 1 high-risk subgroup (n = 1,524). Selection of the high-risk sample involved a risk-prioritization procedure designed to identify individuals with current symptoms and/or a family history of specific disorders.^14^

The assessment phase was performed in multiple visits, in the following order: home interview with parents (1 visit), child assessment with a psychologist (1 or 2 visits), child assessment with a speech therapist (1 or 2 visits), and 1 hospital visit for imaging and blood collection.

From the total sample (N = 2,512), missing data for intelligence and learning problems were handled using listwise deletion. Hence, a subset of 2,240 research participants (862 randomly selected and 1,378 high-risk) with complete intelligence measurements^16^ were included in the present analysis. In this subsample, 1,987 research participants (783 randomly selected and 1,204 high-risk) had complete measurements of learning problems.^17^ Participants with missing intelligence data had lower mean age (9.53 versus 10.37; F1,2510 = 81.28, *p* < .001) than included participants, but did not differ on gender, socioeconomic status, or psychiatric symptoms. Parent informants were mother (91.6%), father (4.4%), or both (4%).

### Positive Attributes Measurement

To measure positive attributes in children and adolescents, we used the Youth Strength Inventory (YSI), a subscale of the Development and Well-Being Assessment (DAWBA).^11^ The YSI is a 24-item scale, divided into 2 blocks of questions addressed to the caregiver. One block focuses on child characteristics, such as if he/she is “lively,” “easy going,” “grateful,” “responsible,” and has a “good sense of humour.” The other block addresses the child’s actions that please others, such as “Helps around the home,” “Well behaved,” “Keeps bedroom tidy,” and “Does homework without reminding.” Each question is answered “No,” “A little,” or “A lot.” A CFA of YSI yielded a 1-factor solution with adequate goodness-of-fit indices (i.e., root mean square error of approximation [RMSEA] 0.057; 90% CI = 0.055–0.059; comparative fit index [CFI] = 0.957; Tucker Lewis Index [TLI] = 0.950; χ^2^ test of model fit = 2201.316; *p* < .001). Composite YSI scores were derived from saved factor scores from the CFA model (Table S1, available online).

### Intelligence Evaluation

For intelligence, we estimated IQ using the vocabulary and block design subtests of the Weschler Intelligence Scale for Children, 3rd edition (WISC-III),^18^ using the Tellegen and Briggs method^19^ and Brazilian norms.^16^, ^20^

### Psychiatric Evaluation

Psychiatric symptoms were evaluated as a continuous variable, using the Strengths and Difficulties Questionnaire (SDQ).^21^ SDQ is a 25-item questionnaire that provides 5 scores of behavioral and emotional symptoms. For the purposes of this study, we excluded “Peer relationships problems” from the SDQ total because of the conceptual overlap among this variable, psychiatric symptoms, and positive attributes. The resulting measure, the SDQ composite (SDQc), includes “Emotional symptoms,” “Inattention/hyperactivity,” and “Conduct problems.”

Psychiatric diagnosis was assessed using the Brazilian Portuguese version^23^ of the DAWBA.^22^ This structured interview was administered to biological parents by trained lay interviewers and scored by trained psychiatrists who were supervised by a senior child psychiatrist.^14^ For the purposes of the propensity score matching (PSM) analysis, we used the DAWBA broad category of “Any psychiatric diagnosis.”

There were low Pearson’s correlations between YSI and IQ (r = 0.105; *p* < .001) and between SDQ and IQ (r = −0.146; *p* ≤ .001). There was a moderate correlation between YSI and SDQc (r = −0.560; *p* ≤ .001).

### Educational Evaluations

Educational evaluations consisted of direct measurement of learning problems in children and by the caregiver’s report of the child’s performance in academic subjects.

Specifically, learning problems were measured by participants’ scores on the School Performance Test (Teste de Desempenho Escolar [TDE]).^17^ The TDE is composed of 2 subtests, decoding (recognition of words isolated from context) and writing (isolated words in dictation). A previous TDE study from our group used latent class analysis (LCA) to identify a cluster of children (18.5% of the sample) with poor decoding and writing skills.^24^ Here, we used membership in this cluster to identify children with learning problems.

Academic performance was measured using the Child Behavior Checklist for ages 6 to 18 (CBCL-School),^25^ completed by the caregiver. The academic subjects assessed were Portuguese or literature, history or social studies, English or Spanish, mathematics, biology, sciences, geography, and computer studies. Each participant was scored as failing, below average, average, and above average. The CFA of CBCL-School = 0.056; 90% CI = 0.048–0.065; CFI = 0.997; TLI = 0.996; χ^2^ test of model fit = 49787.4; *p* < .001). The composite CBCL-School (academic performance) scores were derived from saved factor scores from the CFA model (Table S2, available online).

### Statistical Analysis

We performed a stepwise analysis. We used 2 analytic methods to test the first hypothesis. First, we performed a CFA to investigate if YSI and SDQc items load onto 1 or 2 latent factors. Specifically, we fitted 1-factor, 2-factor, second-order, and bifactor models. (For CFA methods and results, see Supplement 1, available online). Second, we used an LCA to identify groups differing on level of positive attributes. We then used PSM to test whether children differing only in positive attributes (and not on psychiatric diagnosis, symptoms, medication, IQ, age, gender, siblings, socioeconomic status, or parents’ psychiatric diagnoses) differed on school outcomes. Specifically, after PSM, generalized estimating equations (GEE) models were used to test between-group differences in school outcomes. Because school outcomes might vary among the 57 schools, we controlled for cluster effects (random effects) in all statistical tests. The LCA and PSM methods and results are detailed in Supplement 1, available online.

We tested the second hypothesis using univariate models that included 1 independent variable at a time (i.e., YSI, IQ, SDQc), followed by bivariate models that included YSI and IQ or SDQc in the same model without the interaction term, and finally a full model that included the main effects of YSI and IQ or SDQc and the interaction term (i.e., YSI*IQ and YSI*SDQc). To facilitate interpretation, IQ, positive attributes, and psychiatric symptom scores were transformed into standardized units (*z* scores), regressing out the effects of age and gender (using Studentized residuals). Again, study hypotheses were tested using GEE models in SPSS 17 (SPSS Inc., Chicago, IL). We used binary logistic and linear regression models for learning problems and poor academic performance, respectively. Therefore, model estimates (odds ratios [OR] and β) reflect the outcome additive increase for changing 1 standardized unit of the predictors. Interactions were represented graphically using regression surfaces implemented in R (plot3D package^26^). We used marginal effects implemented in Stata version 13 (StataCorp, College Station, TX) to test the significance of the continuous interactions. Marginal effects represent the change in linear prediction (linear regression) and probability (logistic regression) of an outcome for 1 IQ or SDQc standardized unit change when YSI is held constant at different values (–3.5 to 3.5, with 0.5-unit increases). For logistic regression, results were transformed from chances into probabilities to facilitate interpretation. For marginal effects analysis, we used the inverse levels of IQ (IQ * [–1]). For post hoc power analyses of the main models, see Supplement 1, available online.

## Results

### Hypothesis 1

Hypothesis 1 was that positive attributes are empirically discriminable from psychiatric symptoms. CFA indicated that the model with 2 correlated factors showed the best fit indices over the other models (1-factor, second-order, and bifactor models). The model with 2 correlated factors (psychiatric symptoms and positive attributes) showed acceptable goodness-of-fit across indices (RMSEA 0.061, 90% CI = 0.059–0.062, CFI = 0.903, TLI = 0.895, χ^2^ test of model fit = 66086.108, *p* < .001) as the model with 1 factor provided an unacceptable fit to the data according to 2 of 3 fit indices (RMSEA = 0.077, 90% CI = 0.076–0.079, CFI = 0.842, TLI = 0.830, χ^2^ test of model fit = 11012.799, df = 689, *p* < .001). The χ^2^ test for difference testing 1-dimensional versus correlated 2-factor models showed advantages of the 2-factor correlated model over the 1-factor model (χ^2^ = 667.338, df = 1, *p* < .0001). Second-order and bifactor models did not converge.

An item-level inspection of information curves from the CFA of the 2-factor correlated model showed that YSI and SDQc provide information in different areas of a common metric (i.e., YSI is better at discriminating among typically developing children, whereas SDQc is better at discriminating among atypically developing children). Specifically, the mean threshold of SDQc items was –0.19, whereas the mean threshold of YSI items was 0.83 (Figure S1, available online).

LCA indicated that the sample is divided into high (63.2%) and low (36.8%) positive attributes classes (Figure S2, available online). PSM procedures were able to generate 2 groups differing only in positive attributes levels (Figure S3, available online). As predicted, compared to the low YSI group, the high YSI group had lower means on the scale measuring poor academic performance (β = 0.72, 95% CI = 0.65–0.79, *p* < .001). Contrary to our predictions, YSI was not associated with a lower chance of having learning problems (OR = 0.98, 95% CI = 0.73–1.30, *p* = .88).

### Hypothesis 2

Hypothesis 2 was that positive attributes are associated with lower levels of negative educational outcomes independent of intelligence and psychopathology, and through interactions with low intelligence and high levels of psychiatric symptoms that buffer the impact of these 2 variables on negative educational outcomes.

#### Positive Attributes and Intelligence

**Figure 1:**
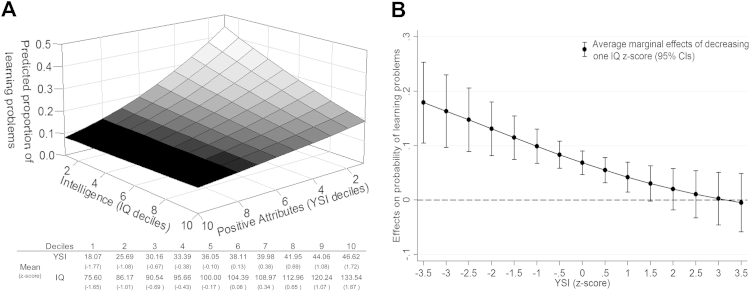
Interaction and marginal effects of intelligence and positive attributes on learning problems. Note: (A) The y-axis represents the probability of learning problems by deciles of intelligence (x-axis) and positive attributes (z-axis). (B) The y-axis represents the probability of learning problems (defined in the text), quantified by the average marginal effect of decreasing 1 IQ *z* score (black dots with CIs) at each Youth Strengths Inventory (YSI) *z* scores (x-axis).

First we analyzed the associations of IQ and YSI on each outcome variable (Table 1). In both univariate and bivariate models, higher YSI and IQ were associated with lower chances of learning problems and lower levels of poor academic performance. For poor academic performance, the associations with IQ and YSI were independent of each other (Table 1, model 3). For learning problems, there was a significant interaction between YSI and IQ, such that the association of intelligence on learning problems was moderated by children’s positive attributes (Table 1, model 3 and Figure 1A). Marginal effect analysis revealed that decreasing levels of IQ were significantly associated with higher probabilities of learning problems for individuals with YSI less than 1.5 *z* score, but not for those with YSI greater than or equal to 1.5 *z* score (Figure 1B). The strength of the association between levels of intelligence and learning problems decreases as a function of increasing levels of positive attributes. For example, at a YSI of –3.5 *z* score, the probability of learning problems increases 17.90% (95% CI = 10.46%–25.33%, *p* < .001) for each IQ standardized unit decrease. At a YSI of 1 *z* score, the probability of learning problems increases 4.21% (95% CI = 1.50–6.93, *p* = .002) for each IQ standardized unit decrease (Figure 1B). Importantly, when the YSI is greater than or equal to 1.5 *z* score, the associations between IQ and learning problems are nonsignificant (Figure 1B), suggesting that high levels of positive attributes buffer the negative impact of low intelligence on learning problems.

#### Positive Attributes and Psychiatric Symptoms

**Figure 2:**
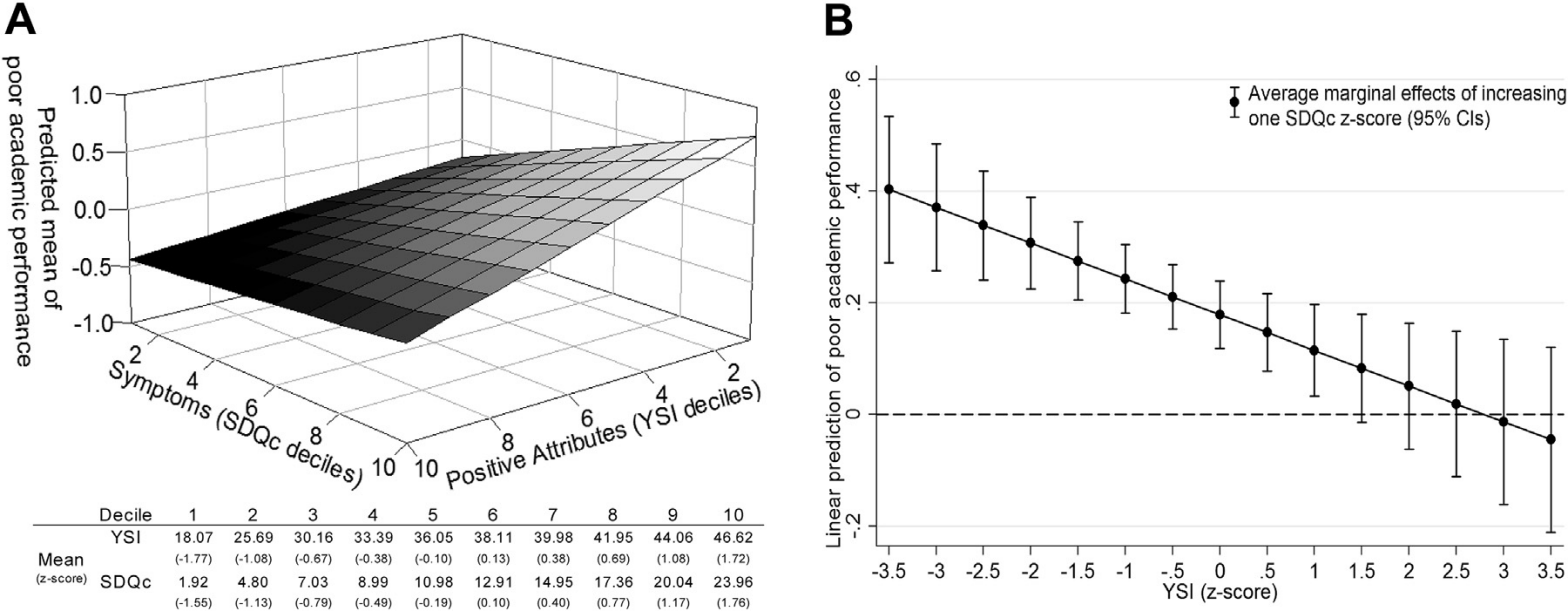
Interaction and marginal effects of psychiatric symptoms and positive attributes on poor academic performance. Note: (A) The y-axis represents the mean of poor academic performance by deciles of psychiatric symptoms (x-axis) and positive attributes (z-axis). (B) The y-axis represents the linear prediction of poor academic performance (defined in the text), quantified by the average marginal effect of increasing 1 composite of Strengths and Difficulties Questionnaire (SDQc; defined in the text) *z* score (black dots with CIs) at each Youth Strengths Inventory (YSI) *z* scores (x-axis).

Finally, we investigated the effect of psychiatric symptoms (SDQc) on school outcomes, again in univariate and bivariate models with child positive attributes (YSI) (Table 2). In the univariate model, higher SDQc was associated with higher levels of negative educational outcomes (Table 2, model 1). In the bivariate models, both YSI and SDQc were significantly associated with learning problems and academic performance (Table 2, model 2). For learning problems, associations with SDQc and YSI were independent (Table 2, model 3). However, for poor academic performance, there was a significant interaction between YSI and SDQc, revealing that the association of psychiatric symptoms on performance in academic subjects is moderated by children’s positive attributes (Table 2, model 3 and Figure 2A). Marginal effect analysis revealed that increasing levels of psychiatric symptoms were significantly associated with poorer academic performance for children and adolescents with YSI less than 1.5 *z* score, but not for those with YSI greater than or equal to 1.5 *z* score (Figure 2B). The strength of the association between levels of psychiatric symptoms and poor academic performance decreases as a function of increasing levels of positive attributes. For example, at a YSI of –3.5 *z* score, linear prediction of poor academic performance increases 0.403 *z* score (95% CI = 0.272–0.534, *p* < .001) for each SDQc standardized unit increase. At a YSI of –1 z score, linear prediction of poor academic performance increases 0.115 *z* score (95% CI = 0.033–0.197, *p* = .007) for each SDQc standardized unit increase (Figure 2B). At YSI greater than or equal to 1.5 *z* score, the association between SDQc and poor academic performance is nonsignificant, suggesting that high levels of positive attributes buffer the negative impact of psychiatric symptoms on academic performance (Figure 2B).

As a post hoc analysis, we ran a second CFA for YSI, excluding items that could overlap with school outcomes (“Keen to learn,” “Good at school work,” “Does homework without needing to be reminded”). A good model fit remained (RMSEA = 0.057, 90% CI = 0.055–0.060; CFI = 0.961, TLI = 0.955, χ^2^ test of model fit = 1681.197, *p* < .001). We re-ran all of the regressions using YSI scores without school items and found the same main effects and interactions described above. Also, for each model, 3-way interactive models among YSI, SDQc, and IQ were nonsignificant, as were interactions with gender.

## Discussion

In this school-based community sample, we first used 2 analytic approaches to investigate the validity of the children’s positive attributes construct. In particular, we were interested in ascertaining the extent to which positive attributes and psychiatric symptoms are distinct constructs. First, CFA showed that a model with 2 correlated factors (positive attributes and psychiatric symptoms) fit better than a unidimensional model. Second, propensity score analysis showed that, even after matching participants for psychiatric symptoms, psychiatric disorders, intelligence, and other potential confounders, children with low positive attributes had worse performance in academic subjects than those with high positive attributes. Finally, we found that positive attributes are associated with better educational outcomes both independent of intelligence and psychiatric symptoms, and by buffering associations among low intelligence, high levels of psychiatric symptoms, and negative educational outcomes.

Consistent with other studies,^11^, ^12^ our results suggest that positive attributes in children are not merely the absence of psychopathology. Although the measurement of psychiatric symptoms might characterize developmental disruptions in children with high levels of psychopathology, the measurement of positive attributes might improve the characterization of behavioral and emotional variability within the normal range, adding incremental health risk prediction.^11^, ^27^ This may explain why positive attributes can predict the risk for later psychiatric disorders in healthy children, beyond predictions based on baseline psychiatric symptoms.^11^ In addition, our PSM results revealed that, in groups matched on other relevant characteristics, children high in positive attributes have better academic performance than those low in positive attributes. This is consistent with findings by Krapohl *et al.*,^28^ who observed that academic performance was predicted not only by intelligence but also by personality traits and well-being. Hence, the CFA and PSM analyses supported the validity of the positive attributes construct by improving behavioral characterization and prediction of academic performance.

Most studies examine the predictive value of 1 variable alone—positive attributes,^11^, ^12^, ^29^, ^30^ intelligence,^4^, ^31^ or psychiatric symptoms^32^, ^33^—without investigating interactions. In agreement with previous studies, we found that intelligence, psychiatric symptoms, and positive attributes did indeed have independent associations with educational outcome. However, our study indicates that these variables also interact. Previous studies suggest that early interventions designed to improve noncognitive abilities in disadvantaged children have a brief impact on IQ but longer-lasting effects on school attainment and employment.^33^ Our results suggest that these lasting effects may result from the impact of noncognitive abilities (i.e., positive attributes) on learning. Specifically, based on our findings, it is reasonable to hypothesize that children with low IQ would show particularly marked benefit from early interventions that increase positive attributes, as the impact of low IQ on learning problems is buffered by positive attributes. Also, an association between high positive attributes and lower psychiatric symptoms has been reported,^11^ and interventions that improve such noncognitive skills in childhood appear to be associated with decreased psychiatric symptoms later in life.^33^, ^34^ Although our results are consistent with these previous studies, our study findings also reveal that, with respect to academic performance, the positive effects of noncognitive abilities might be particularly important in highly symptomatic children, as well as in those with low intelligence. This is especially important, given that mental health in adolescence predicts later educational and occupational attainment, rather than background economic and educational status.^35^

The interactions that we observed among positive attributes, intelligence, and psychiatric symptoms are consistent with developmental theories that focus on adaptive human characteristics.^36^ In particular, the theory of human skills formation of Heckman *et al.*^1^, ^7^, ^37^ is well suited to explain the present findings, as it predicts interactions among cognitive skills, noncognitive skills, and health.^37^ As we observed, positive attributes interact with intelligence and psychiatric symptoms to have an impact on school learning and performance in children and adolescents, suggesting mechanisms by which these variables can affect adult outcomes, including educational attainment, employment, crime, and health.^1^ The interactions found in our study further suggest that remediation of single-domain deficits in a developing child could be important not only for that specific domain but also to potentiate other facets of behavioral function. Considering the work of Vidal-Ribas *et al.*^11^ and ours, it is plausible to suggest a “noncognitive reserve mechanism” through which positive attributes decrease the odds of developing psychopathology and educational impairments, similar to the cognitive reserve hypothesis, which proposes that cognitive function acts as a buffer against the development of psychopathology.^31^

Some limitations need to be considered, to interpret our findings properly. First, as this is a cross-sectional study, the possibility of reverse causality (i.e., school factors influencing positive attributes, intelligence, and symptoms) cannot be ruled out. However, a previous longitudinal study on positive attributes^11^ reported larger effects for positive attributes on psychopathology than those reported here. Second, although PSM minimizes the role of potential confounding factors, unobserved variables might introduce residual confounding effects on the associations between YSI and school outcomes and decrease the effect size of positive attributes on reported associations. Third, apart from learning problems, which were measured by a standardized test, other child characteristics and outcomes were assessed by parental report, which may have led to effect overestimation. Further studies should include other sources of information such as school reports, test scores, and teacher reports. Fourth, this study was carried out in a community sample of a single country, and the results may not generalize to other cultures.

Taken together, our study provides further validity for the positive attributes construct, and suggests that positive attributes may interact with intelligence to predict learning problems and with psychiatric symptoms to predict academic performance. Importantly, the deleterious associations of psychiatric symptoms and low intelligence are buffered by children’s positive attributes. Further studies should focus on understanding the mechanisms mediating these interactions, and on testing mechanistically informed interventions designed to increase positive attributes, particularly in children with psychiatric symptoms and/or low intelligence.

## Figures and Tables

**Table 1:** Univariate, Bivariate, and Interactive Models of Positive Attributes and Intelligence on School Outcomes

	*z* Score^a^	Learning Problems	Poor Academic Performance
OR (LB – UB)	*β* (LB – UB)
Model 1 (univariate)	YSI	0.78∗∗∗ (0.70 to 0.87)	–0.31∗∗∗ (–0.34 to –0.27)
IQ	0.60∗∗∗ (0.52 to 0.68)	–0.22∗∗∗ (–0.26 to –0.18)
Model 2 (bivariate)	YSI	0.81∗∗∗ (0.73 to 0.91)	–0.29∗∗∗ (–0.32 to –0.25)
IQ	0.61∗∗∗ (0.53 to 0.70)	–0.19∗∗∗ (–0.23 to –0.15)
Model 3 (interactive)	YSI	0.86∗ (0.76 to 0.97)	–0.28∗∗∗ (–0.32 to –0.25)
IQ	0.62∗∗∗ (0.55 to 0.71)	–0.19∗∗∗ (–0.22 to –0.15)
YSI∗IQ	1.16∗ (1.02 to 1.32)	0.02 (–0.02 to 0.06)

Note: For learning problems and poor academic performance, outcomes were defined in the text. β = regression coefficient β; LB = lower bound; OR = odds ratio; UB = upper bound; YSI = Youth Strengths Inventory.

a The first *z* score was used as reference for each independent variable, and estimates reflect the additive OR or β increase for changing 1 *z* score.

∗ *p* ≤ .05; ∗∗*p* ≤ .01; ∗∗∗*p* ≤ .001.

**Table 2:** Univariate, Bivariate, and Interactive Models of Positive Attributes and Psychiatric Symptoms on School Outcomes

	*z* Score^a^	Learning Problems	Poor Academic Performance
OR (LB – UB)	β (LB – UB)
Model 1 (univariate)	YSI	0.78∗∗∗ (0.70 to 0.87)	–0.31∗∗∗ (–0.34 to –0.27)
SDQc	1.27∗∗∗ (1.14 to 1.42)	0.30∗∗∗ (0.26 to 0.34)
Model 2 (bivariate)	YSI	0.84∗ (0.73 to 0.96)	–0.20∗∗∗ (–0.25 to –0.16)
SDQc	1.15∗ (1.00 to 1.32)	0.19∗∗∗ (0.14 to 0.23)
Model 3 (interactive)	YSI	0.83∗∗ (0.72 to 0.95)	–0.20∗∗∗ (–0.25 to –0.16)
SDQc	1.18∗ (1.02 to 1.35)	0.18∗∗∗ (0.14 to 0.22)
YSI∗SDQc	1.10 (0.98 to 1.24)	–0.06∗∗∗ (–0.10 to –0.03)

Note: For learning problems and poor academic performance, outcomes were defined in the text. β = regression coefficient β; LB = lower bound; OR = odds ratio; SDQc = composite of Strengths and Difficulties Questionnaire (defined in the text); UB = upper bound; YSI = Youth Strengths Inventory.

a The first *z* score was used as reference for each independent variable, and estimates reflect the additive OR or β increase for changing 1 *z* score.

∗ *p* ≤ .05; ∗∗*p* ≤ .01; ∗∗∗*p* ≤ .001.
