# Supplementary material for: Positive Attributes Buffer the Negative Associations Between Low Intelligence and High Psychopathology With Educational Outcomes
Source: J Am Acad Child Adolesc Psychiatry. 2016 Jan;55(1):47–53. doi: 10.1016/j.jaac.2015.10.013 (PMC4695393; doi:10.1016/j.jaac.2015.10.013)
Supplement: Supplement 1 [file mmc1.docx]

**Supplement 1**

**SUPPLEMENTARY METHODS, ANALYSIS, AND RESULTS**

**Post Hoc Power Analysis**

Post hoc power analyses were conducted for our main outcomes. For our linear outcomes (academic performance), the observed power for the main effects of Youth Strengths Inventory (YSI) and Strengths and Difficulties Questionnaire composite (SDQc) and for their interaction were >0.99, >0.99 and >0.95 respectively. For our binary outcome (learning problems), observed power for the main effects of YSI and SDQc, and for their interaction, were all >0.99.

**Factor Analysis From YSI and CBCL-School Items**

For all confirmatory factor analysis (CFA), we used delta parameterization and weighted least square using a diagonal weight matrix with standard errors and mean- and variance-adjusted χ^2^ test statistics (WLSMV) estimators using MPLUS 7.1 software (Muthén and Muthén, Los Angeles, California, USA). Model fit parameters were χ^2^ test of model fit, root mean square error of approximation (RMSEA), comparative fit index (CFI), and Tucker Lewis Index (TLI). Values of RMSEA near or below 0.08 represent acceptable model fit, and values lower than 0.06 represent good-to-excellent model fit.^1^ CFI and TLI values near or above 0.90 represent acceptable model fit, while values higher than 0.95 represent a good-to-excellent model fit. Nested models were tested using χ^2^ for differences using the DIFFTEST option.

*YSI:* The YSI is a 24-item scale, divided into two blocks of questions addressed to the caregiver. One block focuses on characteristics of the child, such as if he/she is “lively,” “easy going,” “grateful,” “responsible,” and has a “good sense of humour.” The other block addresses the child’s actions that please others, such as “helps around the home,” “well behaved,” “keeps bedroom tidy,” “does homework without reminding,” and others. All questions have three possible answers: “No,” “A little,” and “A lot.” The CFA of YSI using a one-factor solution resulted in adequate goodness-of-fit indexes in our sample, converging to a single factor denominated “positive attributes” (see main text). The composite YSI scores were derived from saved factor scores from the CFA model (Table S1).

*CBCL-School Items:* For academic performance, the CFA of CBCL-School using one-factor solution resulted in adequate goodness-of-fit indexes in our sample (see main text). The composite CBCL-School (academic performance) scores were derived from saved factor scores from the CFA model (Table S2).

**Testing if YSI and SDQc Are Overlapping Constructs**

CFA models, including the YSI and SDQc, were used to test whether the two scales assess the same underlying latent construct. The category threshold indicates the expected value of the latent factor at which there is a > 50% probability of endorsing a given category. The mean threshold for each item was computed as the item location on the severity continuum in order to inform the location of the latent trait in which items were more informative.

CFA models were run to test whether the two scales assess the same underlying latent construct. We fitted a one-factor model (all items loading into a general component), a correlated two-factor model with SDQc items loading onto a psychiatric symptoms dimension, and YSI items loading onto a positive attributes dimension; a second-order model, with psychiatric symptoms and positive attributes loading onto one higher order factor; and a bifactor model, with all items loading into a general factor and residuals loading onto two specific factors: psychiatric symptoms and positive attributes. The model with one factor provided an unacceptable fit to the data according to two out of three fit indexes (see main text), and the model with two correlated factors (psychiatric symptoms and positive attributes) showed acceptable goodness-of-fit in practically all indices (see main text). χ^2^ test for difference testing one-dimensional vs. correlated two-factor models showed advantages of the two-factor correlated model over the one-factor model (χ^2^=667.338, df=1, *p*<.0001). Second-order and bifactor models were not identified.

An item-level inspection of information curves from CFA of the two-factor correlated model showed that YSI and SDQc provide information in different areas of a common metric (i.e*.*, YSI is better at discriminating among typically developing children, while SDQc is better at discriminating among atypically developing children). Specifically, the mean threshold of SDQc items was -0.19, whereas the mean threshold of YSI items was 0.83 (Figure S1).

**Propensity Score Matching Methods**

As a stringent test of discriminant validity, we used propensity score matching^2^ to verify whether associations between a child’s positive attributes and school outcomes are independent of intelligence, psychopathology, and other potential confounders. The analyses were conducted in R, using the Propensity Score Matching (PSM)^3^ and MatchIt^4^ packages from R-project.

Before the PSM procedure, a latent class analysis (LCA) was performed to create empirically derived groups with different levels of positive attributes (YSI score). This analysis was conducted in MPLUS 7.1 (Muthén and Muthén, Los Angeles, California, USA). A solution with two classes (FP=97, Loglikelihood=-44513.83, AIC=89221.66, IC=89787.02, ssaBIC=89478.82) showed a high entropy (0.925) and divided the sample into high positive attribute (63.2%) and low positive attribute (36.8%) classes (Figure S2). A solution with three classes showed an intermediate group with moderate level of positive attributes, while one with four classes showed overlapping classes with no discrimination. A two-class solution was selected to maximize sample size and because of the higher entropy level.

We used the nearest neighbour method for the PSM analysis, with a caliper of 0.25 (i.e., the largest allowable difference in propensity score for matched participants was 25%). Before and after matching, we used a measure of standardized bias to assess the balance of the covariates. Standardized differences of means <0.20 are acceptable, and differences <0.10 are considered negligible.

The PSM procedure selected a total of 671 children with low positive attributes who were matched 1:1 with children with high positive attributes as described in the Method section. By this method, we were able to successfully reduce the magnitude of differences (standardized bias) between children with high and low positive attributes. The mean standardized bias for all covariates is shown in Figure S3.

**References:**

1. Hu L, Bentler PM. Cutoff criteria for fit indexes in covariance structure analysis: Conventional criteria versus new alternatives. *Struct Equ Model Multidiscip J*. 1999;6:1-55.

2. Heckman JJ, Ichimura H, Todd P. Matching As An Econometric Evaluation Estimator. *Rev Econ Stud*. 1998;65:261-94.

3. Bousgaard Mortensen S, Klim S. *PSM: Non-Linear Mixed-Effects Modelling Using Stochastic Differential Equations*. http://www.imm.dtu.dk/psm. Published September 10, 2013. Accessed August 1, 2014.

4. Ho D, Imai K, King G, Stuart EA. MatchIt: Nonparametric Preprocessing for Parametric Causal Inference. *J Stat Softw*. 2011;42:1-28.

*
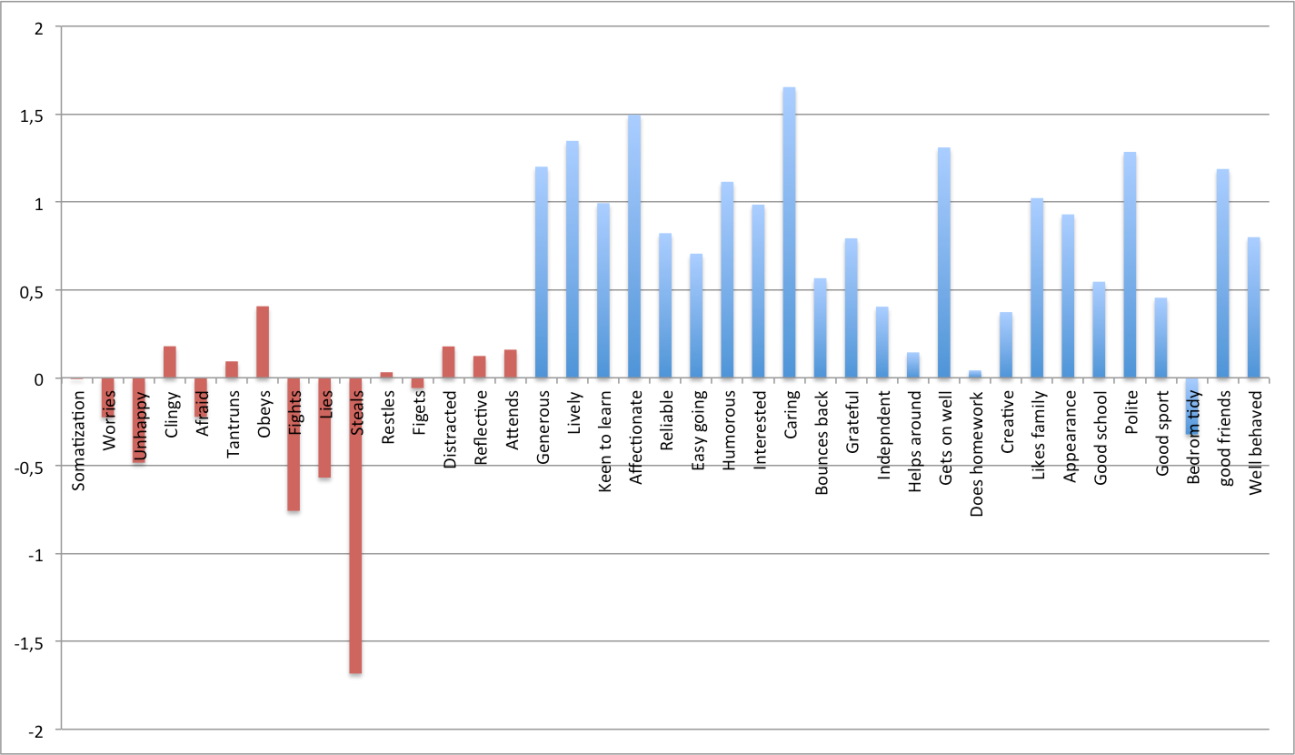
*

**Figure S1:** Standardized average thresholds of each item of the Strengths and Difficulties Questionnaire composite (SDQc) items (in red), and Youth Strengths Inventory (YSI) items, in blue.


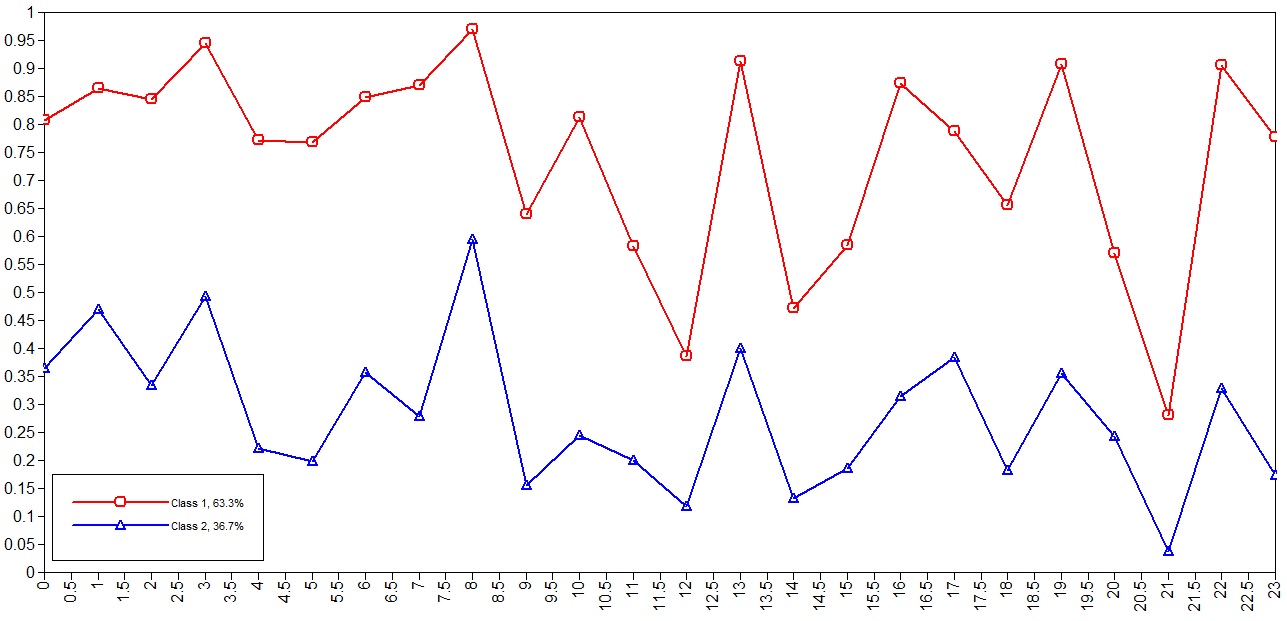


**Figure S2:** Higher Youth Strengths Inventory (YSI) score class is shown in red, while lower YSI score class is shown in blue. Note: Graph represents the chance of endorsement (Y axis) of each item of the YSI (X axis).


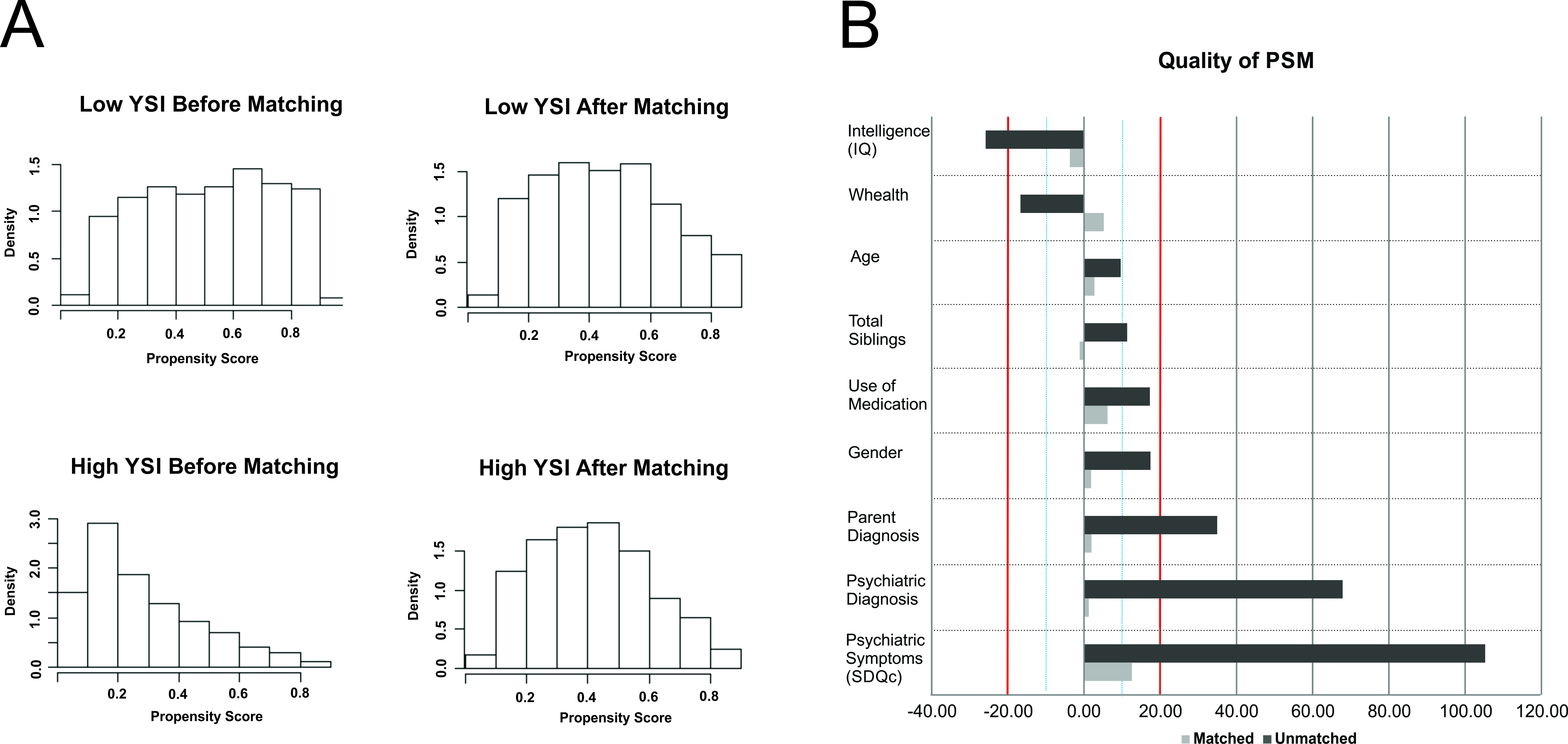


**Figure S3:** (A) Histograms of propensity score matching of high Youth Strengths Inventory (YSI) and low YSI before and after matching and (B) standardized bias (%) of covariates before and after matching. Note: Blue line represents 10% standardized bias limit; below the blue line was considered negligible. Red line represents 20% limit of standardized bias; below was considered acceptable. PSM = propensity score matching.

| **Table S1**. Confirmatory Factor Analysis of Youth Strengths Inventory | | | | |
| --- | --- | --- | --- | --- |
|  | **Factor Loadings** | **SE** | **Thresholds** | |
|  |  |  | **B1** | **B2** |
| Generous | 0.598 | 0.019 | -2.031 | -0.371 |
| Lively | 0.620 | 0.019 | -2.117 | -0.579 |
| Keen to learn | 0.677 | 0.016 | -1.581 | -0.406 |
| Affectionate | 0.753 | 0.016 | -2.222 | -0.770 |
| Reliable and responsible | 0.740 | 0.013 | -1.469 | -0.176 |
| Easy going | 0.746 | 0.012 | -1.264 | -0.148 |
| Good fun, good sense of humour | 0.698 | 0.015 | -1.796 | -0.434 |
| Interested in many things | 0.759 | 0.013 | -1.577 | -0.393 |
| Caring, kind-hearted | 0.777 | 0.018 | -2.343 | -0.965 |
| Bounces back quickly after setbacks | 0.654 | 0.015 | -1.229 | 0.096 |
| Grateful, appreciative of what he gets | 0.761 | 0.012 | -1.324 | -0.263 |
| Independent | 0.535 | 0.017 | -0.954 | 0.145 |
| Helps around the home | 0.438 | 0.020 | -0.852 | 0.563 |
| Gets on well with the rest of the family | 0.762 | 0.015 | -2.024 | -0.597 |
| Does homework without needing to be reminded | 0.514 | 0.018 | -0.478 | 0.393 |
| Creative activities: art, acting, music, making things | 0.571 | 0.017 | -0.903 | 0.156 |
| Likes to be involved in family activities | 0.740 | 0.014 | -1.609 | -0.436 |
| Takes care of his appearance | 0.577 | 0.019 | -1.502 | -0.357 |
| Good at school work | 0.618 | 0.016 | -1.139 | 0.046 |
| Polite | 0.779 | 0.014 | -2.031 | -0.539 |
| Good at sport | 0.458 | 0.02 | -1.036 | 0.125 |
| Keeps his bedroom tidy | 0.53 | 0.018 | -0.23 | 0.874 |
| Good with friends | 0.773 | 0.013 | -1.871 | -0.505 |
| Well behaved | 0.763 | 0.012 | -1.46 | -0.140 |
| **Note:** Errors of the following items were correlated in the model: Good at school with Keen to learn (r=0.278), Does homework without need to be reminded (r=0.399), and Creative activities (r=0.212); Good fun/humour with Lively (r=0.353); Interested in many things with Keen to learn (r=0.251); Caring/Kind-hearted with Affectionate (r=0.215) and Generous (r=0.204); Keeps his/her bedroom tidy with Helps around (r=0.272) and Does homework without need to be reminded (r=0.208); Well behaved with Polite (r=0.178); Affectionate with Generous (r=0.223); Creative activities with Does homework without need to be reminded (r=0.249). SE = standard error. | | | | |

| **Table S2.** Confirmatory Factor Analysis of Performance in Academic Subjects From Child Behavior Checklist | | | | | |  |
| --- | --- | --- | --- | --- | --- | --- |
|  | **Factor Loadings** | **SE** | **Thresholds** | | | |
|  |  |  | **B1** | **B2** | **B3** | |
| Portuguese/Literature | 0.876 | 0.006 | -1.421 | -0.779 | 0.898 | |
| History/Social Studies | 0.904 | 0.005 | -1.563 | -0.978 | 0.999 | |
| Mathematics | 0.69 | 0.012 | -1.484 | -0.732 | 0.721 | |
| Science | 0.887 | 0.005 | -1.610 | -1.023 | 0.978 | |
| Geography | 0.928 | 0.004 | -1.591 | -1.034 | 1.034 | |
| English/Spanish | 0.735 | 0.015 | -1.484 | -0.940 | 0.957 | |
| Computer course | 0.662 | 0.024 | -1.844 | -1.429 | 0.696 | |
| Biology | 0.888 | 0.015 | -1.259 | -0.891 | 1.091 | |
| **Note:** Errors of the following items were correlated in the model: English/Spanish with biology (0.198), computer course with biology (0.170), English/Spanish with computer course (0.202). SE = standard error. | | | | | | |
